# Supplementary figures and images for: SARS-CoV-2 Nucleocapsid Protein Induces Tau Pathological Changes That Can Be Counteracted by SUMO2
Source: Int J Mol Sci. 2024 Jun 28;25(13):7169. doi: 10.3390/ijms25137169 (PMC11241313; doi:10.3390/ijms25137169)

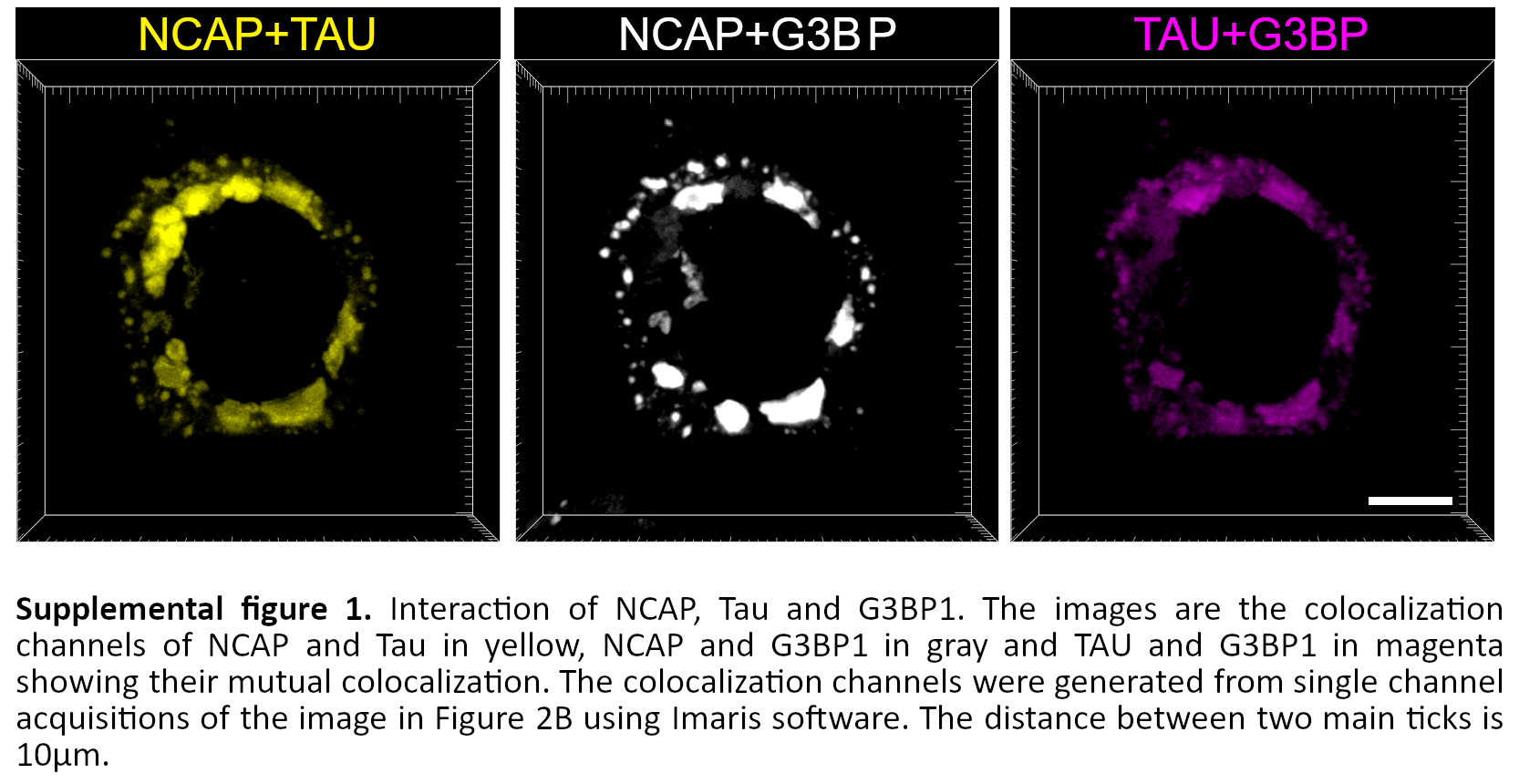

Supplement: Supplementary file 1 [file ijms-25-07169-s001.zip › ijms-3062114-supplementary.TIFF]
